# The Complete Genome Sequence of *Yersinia* *pseudotuberculosis* IP31758, the Causative Agent of Far East Scarlet-Like Fever

**DOI:** 10.1371/journal.pgen.0030142

**Published:** 2007-08-31

**Authors:** Mark Eppinger, M. J Rosovitz, Wolfgang Florian Fricke, David A Rasko, Galina Kokorina, Corinne Fayolle, Luther E Lindler, Elisabeth Carniel, Jacques Ravel

**Affiliations:** 1 J. Craig Venter Institute/The Institute for Genomic Research, Microbial Genomics, Rockville, Maryland, United States of America; 2 *Yersinia* Research Unit, Institut Pasteur, Paris, France; 3 Department of Defense, Global Emerging Infections Surveillance and Response System, Silver Spring, Maryland, United States of America; University of Toronto, Ontario, Canada

## Abstract

The first reported Far East scarlet-like fever (FESLF) epidemic swept the Pacific coastal region of Russia in the late 1950s. Symptoms of the severe infection included erythematous skin rash and desquamation, exanthema, hyperhemic tongue, and a toxic shock syndrome. The term FESLF was coined for the infection because it shares clinical presentations with scarlet fever caused by group A streptococci. The causative agent was later identified as *Yersinia pseudotuberculosis,* although the range of morbidities was vastly different from classical pseudotuberculosis symptoms. To understand the origin and emergence of the peculiar clinical features of FESLF, we have sequenced the genome of the FESLF-causing strain Y. pseudotuberculosis IP31758 and compared it with that of another Y. pseudotuberculosis strain, IP32953, which causes classical gastrointestinal symptoms. The unique gene pool of Y pseudotuberculosis IP31758 accounts for more than 260 strain-specific genes and introduces individual physiological capabilities and virulence determinants, with a significant proportion horizontally acquired that likely originated from Enterobacteriaceae and other soil-dwelling bacteria that persist in the same ecological niche. The mobile genome pool includes two novel plasmids phylogenetically unrelated to all currently reported *Yersinia* plasmids. An *icm/dot* type IVB secretion system, shared only with the intracellular persisting pathogens of the order Legionellales, was found on the larger plasmid and could contribute to scarlatinoid fever symptoms in patients due to the introduction of immunomodulatory and immunosuppressive capabilities. We determined the common and unique traits resulting from genome evolution and speciation within the genus *Yersinia* and drew a more accurate species border between Y. pseudotuberculosis and *Y. pestis.* In contrast to the lack of genetic diversity observed in the evolutionary young descending Y. pestis lineage, the population genetics of Y. pseudotuberculosis is more heterogenous. Both Y. pseudotuberculosis strains IP31758 and the previously sequenced Y. pseudotuberculosis strain IP32953 have evolved by the acquisition of specific plasmids and by the horizontal acquisition and incorporation of different genetic information into the chromosome, which all together or independently seems to potentially impact the phenotypic adaptation of these two strains.

## Author Summary

We have analyzed the genome sequence of a Y. pseudotuberculosis isolate responsible for Far East scarlet-like fever (FESLF). FESLF leads to severe clinical manifestations, including scarlet-like skin rash, from which this illness gets its name, and, most importantly, a toxic shock syndrome not seen in common *pseudotuberculosis* infections. The aim of this study was to catalogue the genomic inventory and get insights in the origin and emergence of this disease. The genus *Yersinia* comprises two other pathogens that cause worldwide infections in humans and animals: Y. enterocolitica, like *Y. pseudotuberculosis,* causes gastrointestinal disorders*,* while Yersinia pestis is the causative agent of plague, also known as the “Black Death.” By comparing the genome of these three *Yersinia* species, we could identify several unique virulence determinants, many of which are known to trigger and modulate the host immune system response and may be intimately associated with the severe and atypical FESLF clinical presentations. We have shown that the reductive gene loss process that Y. pestis has undergone since emerging from the enteric pathogen Y. pseudotuberculosis is not as extensive as originally thought. On the other hand, our analysis indicates that gene acquisition is a major factor that influenced Y. pseudotuberculosis genome evolution.

## Introduction

Yersinia pseudotuberculosis is a bacterial pathogen that, with Y. pestis and *Y. enterocolitica,* causes worldwide infections in humans [1–4]. Y. pseudotuberculosis serotype O:1b is thought to be the direct evolutionary ancestor of *Y. pestis,* the causative agent of plague [4,5]. While these two species diverged from one another within the last 20,000 y, the Y. pseudotuberculosis and Y. enterocolitica lineages separated between 0.4 and 1.9 million y ago [6]. Y. pseudotuberculosis infections in humans are acquired through the gastrointestinal tract by the ingestion of contaminated food products and result in abdominal pain, fever, and occasionally diarrhea. Pathogenicity has been attributed to several key virulence factors, including the plasmid-borne *Yersinia* outer proteins that are delivered by a type III secretion system, the invasion adhesion molecule (Inv), and the high pathogenicity island (HPI) [1]. Often, Y. pseudotuberculosis isolates from environmental and clinical sources harbor various plasmids ranging in size from 3–125 kb [7], some of which have been linked to pathogenicity [8,9]. In 1959, an epidemic of Y. pseudotuberculosis infections on the Pacific coast of Russia was called Far East scarlet-like fever (FESLF), or scarlatinoid fever [10–17] for its clinical similarities to scarlet fever caused by group A streptococci [18,19]*.* Such atypical infections in Far East Asia are severe, and the clinical presentation includes erythematous skin rash, skin desquamation, exanthema, hyperhemic tongue, and toxic shock syndrome [10,11,18,19]. Y. pseudotuberculosis FESLF symptoms have been linked to the systemic expression of the superantigenic exotoxin Y. pseudotuberculosis–derived mitogen (YPM) [20], as well as the presence of two uncharacterized plasmids, pVM82 and pIB [7,8]. Although no plasmid sequence was available, a 37.5-kb region of pVM82 was experimentally linked to increased immunosuppressive and antiphagocytic capabilities [21]. Here, we report the whole genome sequence analysis of serotype O:1b Y. pseudotuberculosis IP31758 that was isolated in 1966 from the stools of a patient presenting with FESLF in the Primorski region of the former Soviet Union. Intra- and interspecies comparisons with the genomes of the previously sequenced typical non-FESLF–causing Y. pseudotuberculosis strain IP32953 [22], all published Y. pestis genomes [23–26], and the more distantly related Y. enterocolitica strain 8081 [27] were performed in order to identify strain-specific genome characteristics of Y. pseudotuberculosis IP31758 intimately related to the atypical clinical FESLF manifestation. In addition, we tested for their distribution in a panel of geographically and phenotypically diverse Y. pseudotuberculosis and Y. pestis isolates. These analyses resulted in the identification of genetic traits potentially associated with the particular FESLF symptoms and led to a redefined model for the evolutionary history of the group.

## Results/Discussion

### General Genome Features

**Figure 1:**
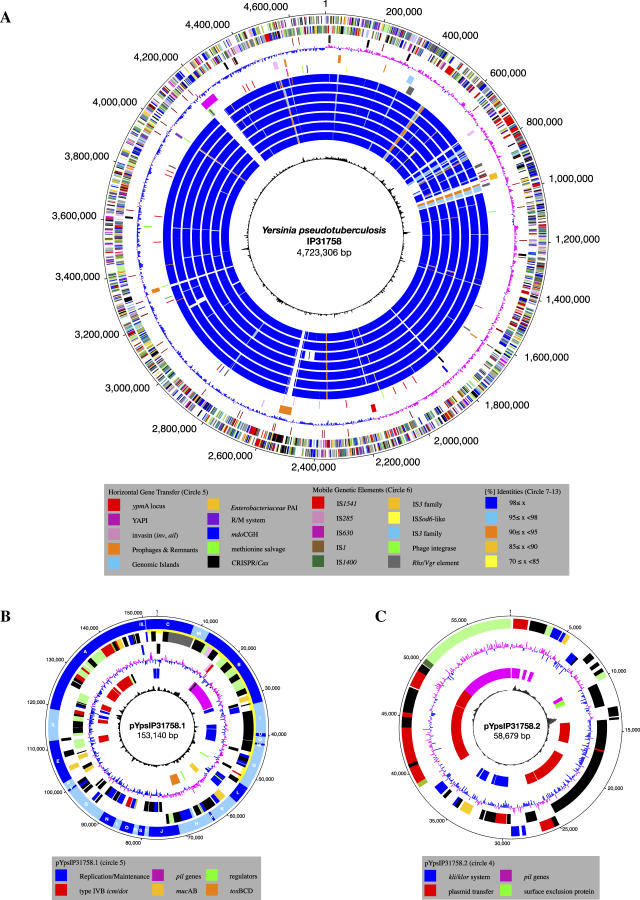
Circular Representation of the Y. pseudotuberculosis IP31758 Genome Chromosome (A), pYpsIP31758.1 (B), and pYpsIP31758.2 (C). (A) Chromosome. Circles from outer to inner circle. (Circle 1–2) Predicted CDSs encoded on the plus (circle 1) and minus strands (circle 2), colored according to the respective TIGR role IDs (http://cmr.tigr.org/tigr-scripts/CMR/RoleIds.cgi). (Circle 3) Noncoding RNAs. Brown, tRNA genes; black, ribosomal rRNAs. (Circle 4) GC skew. (Circle 5) Genomic islands, including the YAPI_IP31758_ pathogenicity island, the Enterobacteriaceae PAI, strain-specific prophage insertions and phage remnants, plasmid-borne regions, and the superantigenic *ypmA* locus. Shared IP31758- and IP32953-specific R/M system with adjacent integrase. (Circle 6) IS elements, transposases, and phage integrases. (Circles 7–13) Comparative analysis of the genomic inventory. Intraspecies comparison of Y. pseudotuberculosis strains IP31758 and IP32953 (circle 7), interspecies comparison of Y. pseudotuberculosis IP31758 with Y. pestis strains CO92 (circle 8), KIM (circle 9), 91001 (circle 10), Nepal516 (circle 11), Antiqua (circle 12), and Pestoides F (circle 13). (Circle 14) Chi square. (B) Plasmid pYpsIP31758.1. (Circle 1) HindIII restriction map. (Circles 2–3) Predicted CDSs encoded on the plus (circle 2) and minus (circle 3) strands, colored according to the respective TIGR role IDs (http://cmr.tigr.org/tigr-scripts/CMR/RoleIds.cgi). (Circle 4) GC skew. (Circle 5) Plasmid regions of interest. (Circle 6) Chi square. (C) Plasmid pYpsIP31758.2. (Circles 1–2) Predicted CDSs encoded on the plus (circle 1) and minus (circle 2) strands, colored according to the respective TIGR role IDs. (Circle 3) GC skew. (Circle 4) Plasmid regions of interest. (Circle 5) Chi square.

The genome of Y. pseudotuberculosis IP31758 consists of a circular chromosome of 4,723,306 bp (Figure 1A) and two novel plasmids called pYpsIP31758.1 (153,140 bp; Figure 1B) and pYpsIP31758.2 (58,679 bp; Figure 1C). Noteworthy, the highly conserved low-calcium response plasmid (lcr) pYV encoding the type III secretion apparatus, which can be found in many but not all Y. pseudotuberculosis and Y. enterocolitica isolates [28–30], was not detected in Y. pseudotuberculosis IP31758. The general genomic features of Y. pseudotuberculosis IP31758 are summarized and compared with those of Y. pseudotuberculosis IP32953 in Table 1. Based on the level of sequence-read coverage in each assembly, it is estimated that the chromosome and the two plasmids are present in equal copy numbers. The combination of these two plasmids has not been reported in any other *Yersinia* strain, and no significant similarity has been found with the known *Yersinia* plasmid sequences from the public databases (Table 1) [22–26,31]. However, the plasmid replication protein RepA (YpsIP31758_B0136) of pYpsIP31758.1 displays 42% amino acid identity to the corresponding gene of the cryptic conjugative plasmid pYptb32953 (pYptb0001) of the previously sequenced Y. pseudotuberculosis strain IP32953 [22]. Y. pseudotuberculosis IP31758 large plasmid, pYpsIP31758.1, was identified as virulence plasmid pVM82, named for its estimated molecular weight (82 kDa) and previously reported in Y. pseudotuberculosis FESLF strains isolated from different areas of the former Soviet Union [7]. The prevalence of this plasmid in FESLF-causing Y. pseudotuberculosis strains and its association to virulence has been experimentally demonstrated [32]. Although no sequence data were available, a HindIII restriction map of pVM82 has been previously published [33]. A thorough comparison of pVM82 HindIII restriction map with that generated in silico from the sequence of pYpsIP31758.1 (153,140 bp) revealed a few discrepancies in the number of restriction fragments and the order in which those were originally assembled (Table S1 and Figure 1B), both of which could be explained by the insufficient resolution of the initial restriction fragment analysis by gel electrophoresis [33]: (1) the in silico HindIII digest of pYpsIP31758.1 resulted in two additional fragments (I [1,678 bp] and II [7,188 bp]), the sizes of which were almost identical to other large fragments and hence would have been impossible to distinguish by gel electrophoresis (fragment P [1,651 bp], fragment I [7,057 bp], and fragment J [7,098 bp]; Figure 1B and Table S1); (2) a small 44-bp fragment (III) was not previously reported; and (3) the size of the largest restriction fragment, measured at 25 kb, was underestimated and is 31,313 bp. Importantly, overall the sequenced plasmid restriction map is in agreement with the published restriction map (Table S1), including the presence of a 37.5-kb region of pVM82 (fragment F), which was experimentally linked to increased immunosuppressive and antiphagocytic capabilities [21]. The updated HindIII restriction map based on the pYpsIP31758.1 plasmid sequence is shown as additional information in the outer circle of Figure 1B.

**Table 1:**
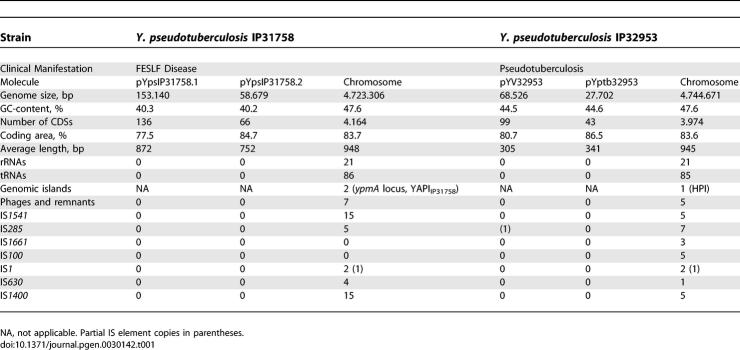
Genomic Features of Y. pseudotuberculosis IP31758 and Comparison with Y. pseudotuberculosis IP32953

Comparative genome sequence analyses between Y. pseudotuberculosis IP32953 and several Y. pestis isolates have shown that Y. pestis has an expanded number of insertion sequence (IS) elements [22]. These IS expansions observed in the Y. pestis lineage had a major impacts on the evolutionary process and speciation by introducing multiple recombinatorial hotspots [22]. Such recombinatorial hotspots account for the intrachromosomal rearrangements (lack of synteny) as well as the reductive evolution (deletion of fragments flanked by IS elements and gene loss due to IS interruption) in the Y. pestis lineage [3,25]. While Y. pseudotuberculosis IP31758 contains a greater number of IS elements than Y. pseudotuberculosis IP32953 (Table 1), both genomes contain far fewer IS elements than *Y. pestis.* A total of six IS families are present in both Y. pseudotuberculosis genomes, although strain-specific IS element distribution patterns and copy numbers are observed (Table 1 and Figures 1A and S1), probably resulting from the process of microevolution (gene loss and acquisition) as well as intrachromosomal IS duplications and translocations, as shown in Y. pestis [34]. The IS elements IS*100* and IS*1661,* both found in all sequenced Y. pestis strains and Y. pseudotuberculosis IP32953 [22,23], were not detected in Y. pseudotuberculosis IP31758 (Table 1). The absence of IS*100* has been previously linked in Y. pseudotuberculosis to sensitivity to pesticin and might indicate a more distant evolutionary and ecological relationship to Y. pestis [35].

### Genome Architecture and Gene Content

**Figure 2:**
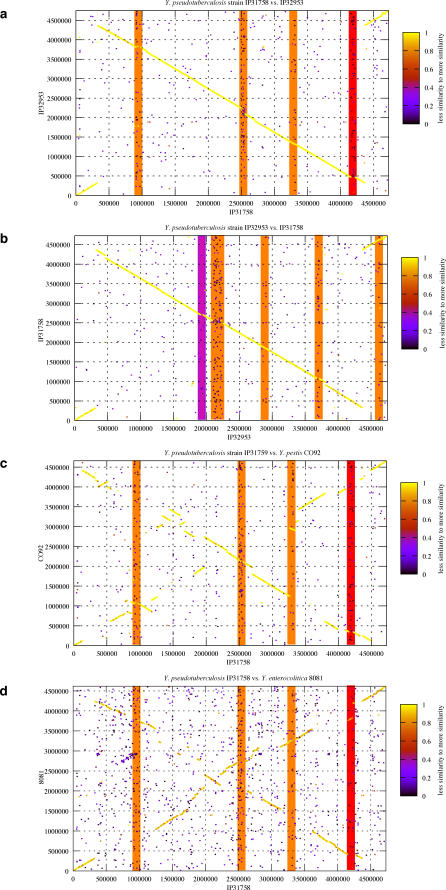
Genome Structure and Synteny (A) Y. pseudotuberculosis IP31758 compared to IP32953. (B) Y. pseudotuberculosis IP32953 compared to IP31758. (C) Y. pseudotuberculosis IP31758 compared to Y. pestis CO92. (D) Y. pseudotuberculosis IP31758 compared to *Y. enterocolitica* 8081. Each protein from the *x*-axis reference genome was queried using BLASTP for its presence in the *y*-axis query genome. For a match, the N-terminal coordinates of both proteins were plotted as *x* and *y*. The color represents the level of similarity of the match expressed by the BLAST score ratio [36]. Prophage insertions are highlighted in orange; the pathogenicity island YAPI_IP31758_ is highlighted in red; and HPI_IP32953_ is highlighted in purple.

Unlike the Y. pestis genome sequences, which display fragmented synteny patterns [25], the two Y. pseudotuberculosis genomes are almost perfectly syntenic and have undergone very little rearrangement (Figure 2A and 2B). A 665-kb inversion encompassing the origin of replication is the only major recombinatorial event that differentiates the two Y. pseudotuberculosis genome sequences as evidenced by the BLAST score ratio analysis (Figure 2) [36]. On the other hand, the synteny at the interspecies level to the genomes of Y. pestis CO92 (Figure 2C) and Y. enterocolitica 8081 (Figure 2D) is partly resolved [26,27]. Similar results were obtained from comparison to all other published Y. pestis genomes. Minor synteny breakpoints are linked to horizontally acquired genomic regions, mainly due to the insertion of prophages, IS elements, and integrons that are specific to each individual Y. pseudotuberculosis strain (Figure 1, circle 5 and Figure S1, circle 5). Sequenced species belonging to the genus *Yersinia* harbor different types and numbers of restriction/modification (R/M) enzyme systems [37]. Noteworthy, our analysis shows that both enteropathogenic Y. pseudotuberculosis strains IP31758 and IP32953 harbor a unique type I R/M system, which is not present in all studied Y. pestis strains, and is composed of three genes, *hsdRSM* (YpsIP31758_3536 to YpsIP31758_3538; Table S6). The implications of this R/M system to Y. pseudotuberculosis genome evolution are still unresolved. Genomic rearrangements do not appear to have been facilitated by intrachromosomal recombination, as they are often flanked by undisrupted housekeeping or hypothetical genes and not by mobile elements or paralogous gene families. Our analysis did not reveal an obvious mechanistic basis for these rearrangements. Compared to the lack of genome-wide synteny found within *Y. pestis,* both sequenced *Y. pseudotuberculosis* strains IP31758 and IP32953 display a high level of genome conservation, which is emphasized by a high degree of nucleotide (nt) sequence identity of more than 95% over 94.8% of the length of the two chromosomes. Such level of nt identity, but conversely with poor synteny, is also observed between Y. pestis and Y. pseudotuberculosis [22], as well as among Y. pestis genome sequences. For the Y. pestis lineage, other than the low degree of synteny, differences on the nt level were attributed to less than 100 single nucleotide polymorphisms [6,23,38].

**Figure 3:**
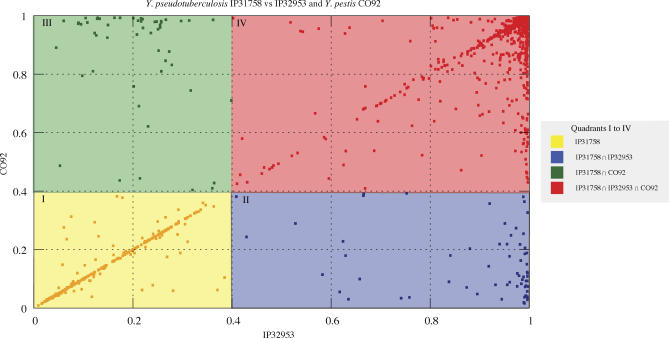
BLAST Score Ratio Analysis of Y. pseudotuberculosis Strains IP31758 and IP32953 and Y. pestis CO92 BLAST score ratios were plotted as *x*,*y* coordinates. Each protein in the reference genome (Y. pseudotuberculosis IP31758) was grouped into four quadrants according to its scores in each of Y. pseudotuberculosis IP32953 and Y. pestis CO92 genomes and colored as follows: yellow (I), unique to IP31758; red (IV), common to all three; blue (II), common between IP31758 and IP32953 but absent in CO92; green (III), common between IP31758 and CO92 but absent in IP32953.

A three-way comparison between both Y. pseudotuberculosis strains IP32953 and IP31758 and Y. pestis CO92 [26] using the BLAST score ratio analysis revealed a high level of protein similarity among all three predicted proteomes with 3,642 conserved gene products and also a more distant phylogenetic relationship of Y. pseudotuberculosis and Y. pestis to Y. enterocolitica (Figure 3 and Table S2) [6]. The availability of a second Y. pseudotuberculosis genome sequence provides the opportunity to refine the set of species-specific genes for Y. pseudotuberculosis from 341 to 67 genes (Table S3), a number that is in agreement with the finding of a subtractive genomic hybridization approach*,* which discovered 112 Y. pseudotuberculosis species-specific small subtractive genomic hybridization fragments with reported insert sizes between 100 to 900 bp [39]. In addition, a total of 265 genes are unique to Y. pseudotuberculosis IP31758 (Table S4) and 289 genes are unique to the previously sequenced Y. pseudotuberculosis IP32953 (Table S5). Examples of such genes include those on the 36-kb *Yersinia* HPI (Figure S1), which is not present in Y. pseudotuberculosis IP31758. The HPI encodes the biosynthetic pathway for the siderophore yersiniabactin and has been shown to play a key role in the systemic spread of the *Yersinia* isolates that harbor this island (all Y. pestis strains and subsets of Y. pseudotuberculosis and Y. enterocolitica) [40]*.*

### Genomic Islands and Pathogenic Potential

**Figure 4:**
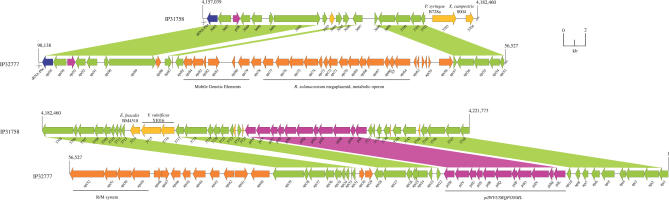
Genomic Architecture of the Y. pseudotuberculosis Pathogenicity Islands YAPI_IP31758_ and YAPI_IP32777_ The YAPI pathogenicity islands are integrated next to a tRNA^Phe^ locus. CDSs shared among both YAPI islands are colored olive with the encoded *pil* (pilin) gene cluster highlighted in magenta, strain-specific CDSs are colored in yellow and orange, and the YAPI-specific phage integrase is colored in blue. The coordinates of the Y. pseudotuberculosis IP32777 are according to [44].

**Figure 5:**
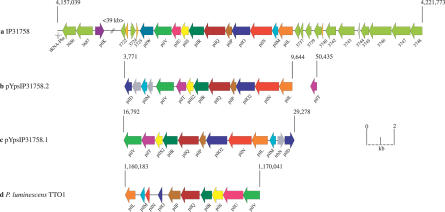
Genomic Architecture and Comparison of the Chromosomal and Plasmid-Borne Type IV *pil* Gene Clusters of Y. pseudotuberculosis IP31758 Y. pseudotuberculosis IP31758 harbors three *pil* (pilin) loci encoded by YAPI_IP31758_ on the core chromosome (A) and on both plasmids pYpsIP31758.2 (B) and pYpsIP31758.1 (C). The chromosomal *pilWVUSRQPONML* locus found within the YAPI_IP31758_ is more similar to that found on the chromosome of P. luminescens TTO1 (D). Corresponding *pil* genes are colored accordingly. Y. pseudotuberculosis IP31758 strain-specific YAPI CDSs are colored orange. Olive CDSs are shared by YAPI_IP31758_ and YAPI_IP32777_.

Multiple regions potentially relevant to pathogenicity appear to have been horizontally acquired and are scattered throughout the Y. pseudotuberculosis IP31758 genome. These regions, comprising prophages, plasmid-like integrons, and genomic islands, are often characterized by a deviating GC content and are often inserted into tRNA genes (Figure 1A). Mobile genetic elements such as those encoding phage-related integrases and IS elements frequently flank these unique regions and result from the specific mode of incorporation. A number of small insertions were most likely horizontally acquired by Y. pseudotuberculosis IP31758 but do not show or have lost their colocalization to mobile elements (Table S6). One example of a horizontally acquired virulence determinant is the *Yersinia* adhesion pathogenicity island (YAPI) that has always been found inserted into one of the two tRNA^Phe^ genes and carries several mobility determinants, such as a phage integrase gene and IS elements (Figure 1A). The YAPI was originally described in Y. pseudotuberculosis serotype I strain IP32777 [41] and is also present in Y. enterocolitica strain 8081 [27,42]. YAPI_IP31758_ is shorter than those previously described. Two large deletions in YAPI_IP31758_ correspond to *api*84*–api*56 and *api*52–*api*40 [41], which code for unrelated metabolic functions and a R/M system, respectively (Figure 4). These deletions account for the difference in size between YAPI_IP31758_ (64 kb) and YAPI_IP32777_ (98 kb). YAPI_IP31758_ contains several unique genes with no assigned function. All known *Yersinia* YAPIs harbor a polycistronic pilin gene cluster *pilWVUSRQPONML.* BLAST analysis of this gene cluster revealed that the best protein similarities outside this yersinial pathogenicity island are found to the respective genes of Photorhabdus luminescens TTO1 (Figure 5D) [43]. The YAPI_IP32777_ cluster has been experimentally shown to be critical for the virulence of Y. pseudotuberculosis IP32777 by mediating adhesion to the respiratory epithelium in a mouse model [41,44]. A comparison of the known YAPI revealed that, while genomic diversity exists in this island, the structure and composition of the *pil* gene clusters are conserved, strengthening its role in pathogenicity (Figure 5). YAPI-encoded surface exposed elements such as pilin might be associated with the severe host immune response observed in patients with FESLF. Supporting the role of pilin components in pathogenicity of Y. pseudotuberculosis IP31758 is the presence of two additional pilin gene clusters on each of the two plasmids. The pYpsIP31758.1-encoded pilin cluster is located in the pVM82 region previously thought to replace the pVM57 F fragment (shown in yellow in Figure 1B; Table S1) and reported to be critical for pathogenicity [8]. The observed altered clinical manifestations as well as the conjugal transfer of pVM82 were attributed to the presence of this pVM82-specific region [21]. Unlike the entire YAPI_IP31758_ *pil* cluster, which is phylogenetically related to that of *P. luminescens,* different parts of the plasmid-borne *pil* clusters are most similar to several other bacterial species, including *Escherichia, Salmonella,* and *Pseudomonas* species, indicating a different phylogenetic origin than those of YAPI_IP31758_. In contrast, Y. pseudotuberculosis IP32953 is YAPI negative and does not produce pilins.

### Superantigenic Toxins in *Y. pseudotuberculosis*

**Figure 6:**
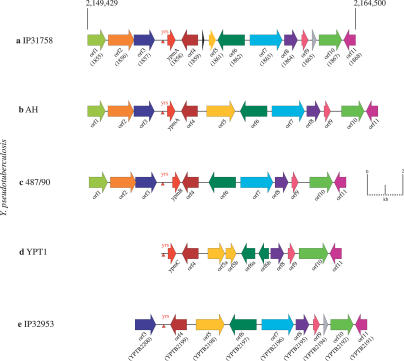
Genomic Architecture of the Superantigenic *ypm* Locus in Y. pseudotuberculosis (A) The scale in bp indicates the genomic location of the Y. pseudotuberculosis IP31758 *ypmA* locus from YpsIP31758_1855 to YpsIP31758_1868 integrated downstream of the *Yersinia* recombination site (*yrs*; red arrow). The Y. pseudotuberculosis IP31758 *ypm*A locus is composed of 12 genes and is compared to the *ypmA*, *B,* and *C* loci of three superantigenic Y. pseudotuberculosis strains AH (B), 487/90 (C), and YPT1 (D), and to the corresponding region of the non-superantigenic strain Y. pseudotuberculosis IP32953 (E). CDSs shared between these loci are colored accordingly with the *ypm* superantigen highlighted in red. The CDS between *orf9* and *orf10* is not predicted in strains AH, 487/90, and YPT1.

Another important virulence-associated factor identified in Y. pseudotuberculosis IP31758 is YPM [45,46]. YPM is a superantigenic toxin that belongs to a class of highly potent immune stimulatory proteins produced by a variety of Gram-positive bacteria and retroviruses [47]. Currently the Y. pseudotuberculosis mitogen is the only known superantigenic toxin identified in Gram-negative bacteria [20,48,49]. The YPM superantigen has been experimentally shown to interfere with the host immune system and is thought to be critical to the pathogenicity of FESLF-causing Y. pseudotuberculosis strains [41,50–53]. YPM may be associated with the particular scarlatinoid fever syndromes because it mediates an uncontrolled host immune system activation [20,54]. This is analogous to the role of superantigens in staphylococcal and streptococcal toxic shock syndromes [18,19]. The similarities in the clinical presentation of scarlet and scarlet-like fever suggest a direct role of YPM in the pathogenesis and the distinct clinical manifestation of Y. pseudotuberculosis isolates causing FESLF. Both superantigenic toxins, YPM and staphylococcal enterotoxin A, are implicated in scarlet-like and scarlet fever and have been shown to interact with multiple eukaryotic signaling pathways in a mouse model [51,52,55]. The *ypm* gene is found in a Y. pseudotuberculosis subgroup isolated predominantly in Far East Asia, and its presence or absence correlates with the different clinical manifestations observed between Far East Asia and Europe [56,57]. Furthermore, high anti-YPM antibody titers reported in patients with FESLF who have systemic infections suggest a direct role of YPM in pathogenicity [50]. In *Y. pseudotuberculosis,* three YPM variants encoded by *ypmA*, *ypmB,* and *ypmC* have been described [54,58] and shown to be integrated downstream of a conserved 26-bp motif known as *Yersinia* recombination site (*yrs*) (Figure 6). This motif is also present in the corresponding locus of the non-superantigenic strain Y. pseudotuberculosis IP32953, which lacks the *ypm* gene and does not produce a superantigen. Comparison of these chromosomal loci showed a strong syntenic organization. In Y. pseudotuberculosis IP31758, this locus is most similar to that of the *ypmA*-containing Y. pseudotuberculosis strain AH, with *ypmA* showing 100% identity at the nt level. The *ypmA* gene is predominantly found in clinical isolates of *Y. pseudotuberculosis* from Far East Asia, while the *ypmB* and *ypmC* loci are associated with environmental and animal isolates [54].

The HPI is present only in a subset of Y. pseudotuberculosis strains and may be lost by spontaneous excision from the chromosome [59,60]. Based on the presence or absence of HPI and *ypmA,* two subgroups can be established that reflect the geographical distribution of *Y. pseudotuberculosis:* The YPMA^+^ HPI^−^ subgroup predominantly comprises far eastern pathogenic types, including those causing FESLF, while the YPMs^−^ HPI^+^ subgroup contains European gastroenteric pathogenic types [57]. The absence of the HPI in Y. pseudotuberculosis IP31758 therefore most likely reflects its divergent phylogenetic branch rather than the secondary loss of this pathogenicity island.

Similar to the staphylococcal enterotoxin A, which is thought to have been acquired through phage infection [61], it has been speculated that the presence of the YAPI_IP31758_-encoded pilus might have favored the acquisition of the *ypmA* locus through phage infection by functioning as an attachment site [44,62]. In support of this hypothesis, a correlation exists between YAPI^+^ strains and YPM^+^ strains in Far East Asian Y. pseudotuberculosis isolates responsible for FESLF [53].

### Relatedness to Enterobacteriaceae Pathogenicity Islands

A 24-kb region (YpsIP31758_0743 to YpsIP31758_0777) characterized by an unusual nt composition exhibits similarity and partial synteny to several reported Enterobacteriaceae pathogenicity-associated islands (PAIs) and is flanked by another copy of the YAPI_IP31758_ phage integrase gene (YpsIP31758_0743, 100% nt identity to YpsIP31758_3686; Figure 1A). This genomic island was reported to be a PAI and is predominantly found in uropathogenic E. coli strains and in several *Shigella* species [63–67]. The presence of the Enterobacteriaceae-related IS*1* and IS*630* elements further supports the phylogenetic origin of this genomic island. This region displays a mosaic composition of phage-like genes encoding integrases and structural components, and the plasmid-borne replication initiation genes *repA* and *repB.* Furthermore, a 21-kb region encoded entirely on the plus strand (YpsIP31758_0312 to YpsIP31758_0327) is similar to and syntenic with other enterobacterial pathogenicity islands [63–67] (Figure 1A). Most of the genes within these two islands encode conserved hypothetical proteins with no assigned functions, and orthologs of these genes are found within enterobacterial PAIs [63–67]. The flanking *Rhs-* and *Vgr*-related loci are often found to be recombinational hotspots in E. coli [68]. However, while these findings could suggest horizontal transfer, the nucleotide composition of this 21-kb region does not show any unusual pattern, no mobile elements are associated with the island, and the region is conserved in all published *Yersinia* species genome sequences. It is unclear if this island represents an ancient insertion event, is the remnant of a γ-proteobacterial ancestor genome, or has been transferred between *Yersinia* and other *Enterobacteriaceae.*

### Strain-Specific Phage Pattern

Phages have been implicated in the evolution of bacterial pathogens [69], and our analysis indicates that phage infections might have been responsible for the acquisition of several of the genomic islands implicated in FESLF pathogenicity [53]. The genome sequence of Y. pseudotuberculosis IP31758 contains several regions that were identified as prophage or phage remnants (Figure 1A and Tables 1 and S6). A large 41-kb prophage called PhiYpsI has been identified. It is encoded entirely on the minus strand and is inserted into tRNA^Leu-2^, which is part of a tRNA^Leu-2-Cys-1-Gly2^ cluster, resulting in two imperfect direct repeats of 124 bp flanking the insertion site. PhiYpsI appears to be complete and potentially functional. PhiYpsI is similar to Enterobacteriaceae phages previously linked to pathogenicity in *Salmonella* and *Shigella* (Table S6) [70,71]. The 10-kb P2-like phage PhiYpsII is found adjacent to PhiYpsI and is encoded entirely on the plus strand. These two phages appear to have inserted in tandem into the same target tRNA cluster. The PhiYpsII phage coding sequences (CDSs) display homology with CDSs of the large 122-kb phage of Y. pseudotuberculosis IP32953 (Figure S1 and Table S6; YPTB1834–1840, YPTB1741–1743) [22]. The similar target tRNA insertion site of these two phages may argue for the presence of a P2-like phage at this site in the ancestor of these two isolates, despite that PhiYpsII in IP31758 appears to have lost parts of this ancestral phage. Another 14-kb prophage, PhiYpsIII (Figure 1A and Table S6), displays similarity to the Burkholderia cenocepacia phage BcepB1A [72,73]. While the phages and their insertion sites can be identified, most of the CDSs encode hypothetical proteins whose relevance to pathogenicity cannot be evaluated, but is not excluded. Recently, the role of the unstable filamentous phage YpfΦ in the pathogenicity and fitness of Y. pestis was demonstrated [74]. The strain-specific prophage profile of the scarlatinoid and gastroenteric pathogenic strains Y. pseudotuberculosis IP31758 and IP32953 together with their unique gene content could potentially be used for the genotyping of clinical Y. pseudotuberculosis isolates.

### Invasion and Adhesion Genes

Besides the sheer presence or absence of virulence determinants in the Y. pseudotuberculosis strains IP31758 and IP32953, genes of the shared genomic inventory revealed distinct polymorphisms, which may affect the pathogenic potential of the individual strains. Variation in length in their respective sets of adhesion genes may alter the adhesive and invasive capabilities of each Y. pseudotuberculosis strain during infection (Figures 1A and S1). The invasins are mediators of pathogenesis in some *Yersinia* species [75] and confer the ability to invade epithelial cells by binding to integrins, collagen, and fibronectin [76]. The invasin gene *(inv)* has been shown to be important in Y. enterocolitica pathogenesis, but its role in Y. pseudotuberculosis is not fully understood, and it plays no role in Y. pestis in which it is nonfunctional [77,78]. Both sequenced Y. pseudotuberculosis strains also encode the attachment invasion locus *(ail)* protein (YpsIP31758_1160, YPTB2867) [79] and a set of three invasins that show length variation (YpsIP31758_0608 [2,795 aa], YpsIP31758_2329 [941 aa], YpsIP31758_4008 [4,953 aa], YPTB1572 [1,075 aa], YPTB1668 [985 aa], and YPTB3789 [5,623 aa]).

### Plasmid-Encoded Virulence Factors on the Y. pseudotuberculosis IP31758 Plasmids pYpsIP31758.1 and pYpsIP31758.2

**Figure 7:**
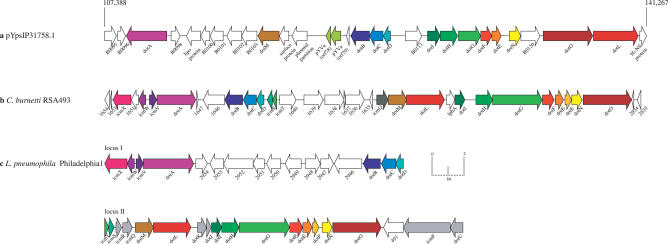
Genomic Architecture of the pYpsIP31758.1-Borne Type IVB Locus and Comparative Analyses to That of C. burnetti RSA493 and L. pneumophila Philadelphia 1 The plasmid pYpsIP31758.1 type IVB locus (A) encodes 28 genes and is compared to that of C. burnetti RSA493 (B) and L. pneumophila Philadelphia 1 (C). The scale in bp indicates the genomic location of the type IVB locus from YpsIP31758_B0095 to YpsIP31758_B0122. Corresponding CDSs involved in type IVB pilus assembly are colored accordingly; species-specific CDSs often found interspersed are colored white. *icm/dot* genes that are unique to C. burnetti and L. pneumophila are colored in dark gray and light gray, respectively.

?>The large plasmid pYpsIP31758.1 (Figure 1B) encodes several factors that could play a role in the pathogenicity of Y. pseudotuberculosis IP31758. A detailed analysis of pYpsIP31758.1 revealed the presence of a type IVB *icm/dot* secretion system (Figure 7). The type IVB *icm/dot* system was initially discovered by examining Legionella pneumophila mutants defective in replication inside the macrophage and in the secretion of distinct effector molecules [80]. This system had previously only been found in *Legionella* and *Coxiella* species [81] and is reported for the first time in the genus *Yersinia*. The Y. pseudotuberculosis IP31758 *icm/dot* locus gene structure is most similar to that of *C. burnetti,* being contained within a single locus, whereas in *L. pneumophila,* this type IVB secretion system is comprised of two separate loci (Figure 7). In addition, the presence of a phage-like integrase (YpsIP31758_B0092) within this cluster may indicate the acquisition in Y. pseudotuberculosis IP31758 via lateral gene transfer. In *Legionella* and *Coxiella,* these secretion systems have only been reported on the chromosome, while Y. pseudotuberculosis IP31758 represents the first instance of an *icm/dot* secretion system encoded on a plasmid as part of the mobile genome pool. The infectious process of both pathogenic *Yersinia* and *Legionellales* is thought to involve a temporary intracellular stage [4]. While this *icm/dot* secretion system is absent in all other sequenced *Yersinia,* it may mediate the intracellular survival of Y. pseudotuberculosis IP31758 in epithelial cells and trigger the host immune system response, both of which are features that may contribute to the unusual scarlatinoid-like clinical presentation [7,82–85]. Aside from DotA, none of the type IVB effector molecules reported for L. pneumophila and C. burnetti [86] are found in the genome of Y. pseudotuberculosis IP31758. However, a number of hypothetical genes found interspersed within the cluster could be potential effector molecule candidates or a unique part of the secretion machinery. Such is the case in *Legionella,* where type IVB gene clusters include distinct hypothetical genes that are found at syntenic locations in different strains and are believed to be involved in the assembly of the secretion machinery. These diversified gene sets appear to be the result of strain-specific adaptation and specialization. This hypothesis is strengthened by the presence of polymorphisms in the secreted effector molecule DotA found in different *Legionella* isolates [87,88]. Y. pseudotuberculosis IP31758 DotA shows aa similarities of 52% and 54% to the respective homologs in L. pneumophila and *C. burnetti.* pYpsIP31758.1 encodes additional features that could potentially play a role in the pathogenicity and overall bacterial fitness of Y. pseudotuberculosis IP31758. This includes a gene cluster *(tox)* similar to that of the biosynthetic operon of the phytotoxin toxoflavin initially described in Burkholderia glumae BGR1. Toxoflavin has been shown to be critical to the pathogenicity and to the overall fitness of B. glumae [89,90]. In addition, a homolog of the *E. coli umuDC* operon that confers UV resistance is present on the plasmid and might contribute to the survival of Y. pseudotuberculosis IP31758 in the environment. The larger plasmid codes for three regulators, the *Yersinia* global negative regulator *(ymoA)* is found adjacent to the *tox* operon, the DNA-binding protein H-NS (YpsIP31758_B0123) found upstream of the type IVB secretion machinery and the hemolysin expression–modulating protein Hha (YpsIP31758_B0044) [91,92]. YmoA is a virulence-modulating regulator that controls multiple virulence-associated genes and is known to interact with the DNA-binding protein H-NS. In Y. pseudotuberculosis IP31758, homologs of *ymoA* are present on both the chromosome (YpsIP31758_3073) and pYpsIP31758.1 (YpsIP31758_B0060), displaying 89% aa similarity. One could speculate on a concerted role for these regulators in modulating plasmid- and chromosome-encoded virulence determinants [93]. pYpsIP31758.1 appears to lack a complete plasmid transfer system; however, such a system is present on the smaller plasmid pYpsIP31758.2 (Figure 1C). The pYpsIP31758.2 transfer system is most similar to that of the *Pseudomonas* species IncP-1beta group pB3 plasmids [94,95], and may also provide the transfer function for the large plasmid. pYpsIP31758.2 is replicated and maintained through a *kil/kor* system. Such a mechanism has not previously been reported in *Yersinia,* nor has the incompatibility surface exclusion protein also found on pYpsIP31758.2 (YpsIP31758_A0016) [96,97].

Together with the chromosomally encoded pathogenicity determinants, the factors present on both pYpsIP31758.1 and pYpsIP31758.2, including the two type IV *pil* gene clusters mentioned previously, might be key to the peculiar clinical presentations of Y. pseudotuberculosis IP31758 FESLF infections.

### Gene Loss and Metabolic Capabilities

Among the 67 Y. pseudotuberculosis species-specific genes in regard to *Y. pestis,* two loci were found to encode metabolic functions. These genes code for the methionine salvage pathway and the *mdoCGH* glucan biosynthetic cluster (Figure 1A). Orthologs have been recently reported to be also present in the distantly related Y. enterocolitica strain 8081 [27]. Osmoregulated periplasmic glucans are intrinsic components of the Gram-negative bacterial envelope. This pathway was initially characterized in Erwinia chrysanthemi osmoprotectant-deficient mutants presenting hypersensitivity to bile salt and antibiotics, reduced enzymatic production, and even complete loss of virulence [98]. *mdoG* and *mdoH* are sufficient for glucan biosynthesis, and deletions in either abolish osmoregulated periplasmic glucans synthesis, whereas *mdoC* is dispensable and thought to succinylate the periplasmic glucan [99,100]. The number of deleterious point mutations observed in the two sequenced Y. pseudotuberculosis isolates suggests *mdoC* might not be functional.

The methionine salvage pathway is present in both Y. pseudotuberculosis strains, IP31758 and IP32953, although it is absent in all sequenced Y. pestis strains [101]. The methionine salvage cycle biochemical pathway maintains methionine levels by recycling methylthioadenosine, a product of the biosynthesis of polyamines such as spermine and spermidine into methionine. The presence of this pathway in the atypical Y. pestis subspecies pestoides F and Y. enterocolitica strain 8081 [27] suggests that this loci has been lost in Y. pestis and was present in the ancestral root of this lineage*.* This hypothesis is strengthened by the absence of deviating GC content or colocalization of mobile genetic elements at this locus that would indicate a recent or ancient acquisition. In addition, *mtnN* (5′-methylthioadenosine/S-adenosylhomocysteine nucleosidase), a component of the pathway located elsewhere in the genome, remains present in all sequenced Y. pestis strains.

### Identification of the IP31758-Specific Genetic Elements That Might Be Associated with the FESLF Clinical Presentation

**Figure 8:**
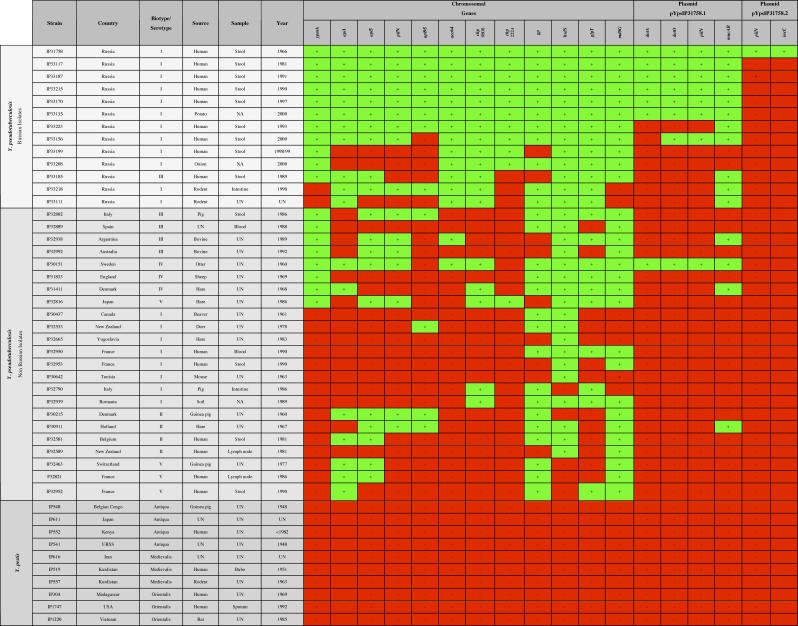
Screening for Unique Y. pseudotuberculosis IP31758 CDSs against Representative Strains of Y. pseudotuberculosis and Y. pestis A panel of 46 geographically and phenotypically diverse Y. pseudotuberculosis (36) and Y. pestis (ten) isolates representing the genetic diversity were screened for the presence (+) or absence (−) of unique chromosomal regions and the plasmid distribution identified in Y. pseudotuberculosis IP31758. UN, unknown; NA, not applicable.

To expand the analysis, a panel of 46 geographically and phenotypically diverse Y. pseudotuberculosis and Y. pestis was screened for the presence of the identified unique chromosomal regions and plasmid content of Y. pseudotuberculosis IP31758 (Figure 8 and Table S6). We attempted to determine those genetic regions that differentiate the gastroenteric pathogenic type Y. pseudotuberculosis strain IP32953 from the FESLF-causing Y. pseudotuberculosis strain IP31758 and might be directly responsible for the peculiar clinical features of FESLF. The occurrence of such genes should be uniform within distinct Y. pseudotuberculosis FESLF isolates, while genes whose presence is variable within strains probably are not related to the clinical FESLF manifestation. The isolates selected encompass *Yersinia* genetic diversity (serotype: I, II, III, IV, V; biotype: Antiqua, Medievalis, Orientalis) and include 11 isolates from the time period of the first reported FESLF epidemic on the east coast of Russia [10] (Figure 8 and Table S6). We also tested for the prevalence of pYV within 12 other Russian isolates used in the study (Carniel et al., personal communication). This analysis revealed that 9 strains (IP33117, IP33215, IP33125, IP33223, IP33156, IP33199, IP33208, IP33218, and IP33185) contained pYV, while 3 isolates (IP33187, IP33170, and IP33111) lack pYV. It is not uncommon for pathogenic *Yersinia* to lose pYV in vitro, in particular when incubated at 37 °C, the temperature used for stool cultures in clinical microbiology laboratories (Figure 8) and used for the isolation of Y. pseudotuberculosis IP31758. Similarly, Y. pseudotuberculosis IP33187 and IP33170, two pYV^−^ isolates, were isolated from the stools of patients with FESLF. Furthermore, a number of Y. pseudotuberculosis and Y. enterocolitica isolates have been reported to be pathogenic while lacking pYV [28–30]. Because of the high sequence similarity between all Y. pseudotuberculosis pYV or Y. pestis pCD plasmids [9], it is unlikely that pYV is responsible for the unique clinical manifestation of FESLF disease, but when present, pYV might contribute to the pathogenic potential of the isolates, such as IP33223 and IP33199. The 36 Y. pseudotuberculosis isolates selected encompassed the main classical serotypes (I to V) found worldwide and included another 12 isolates from Russia, of which eight were isolated from human stools (six of them from patients presenting FESLF symptoms: IP33223, IP33170, IP33187, IP33199, IP33156, and IP33185). The remainder included isolates from wildlife and environmental samples for which no clinical phenotypes were assigned.

Five of the Russian isolates harbored all the loci tested but those of pYpsIP3158.2, suggesting that they are genetically homogeneous. However, a broader diversity was found in the other isolates, some of which known to cause FESLF. This strengthens the findings that the genomic diversity in Y. pseudotuberculosis is greater than originally thought. Interestingly, two Y. pseudotuberculosis strains (IP33208 and IP33199) isolated from stools of patients with FESLF appeared to be lacking three and four of the pYpsIP31758.1 loci tested, respectively. This result might indicate that in these isolates, either the sequence at these loci is missing or divergent from that of pYpsIP31758.1, or the plasmid is lacking. The latter is not supported by previous experiments showing that pVM82 is critical for the pathogenicity of FESLF [8,21]. Overall, the tested markers are restricted to Y. pseudotuberculosis and narrowly distributed in Far East Asian isolates; they might therefore play a role in FESLF pathogenicity [102]. The genetic heterogeneity between Y. pseudotuberculosis isolates in far eastern and western countries is documented in our screening analysis [56,57]. The pattern linked to Y. pseudotuberculosis IP31758 dominates in Far East Asia, and the modern Russian strains still harbor the unique characteristics of the original strain. The superantigenic toxin *ypmA* was found in all FESLF-causing strains as well as in two Russian environmental isolates. However, a PCR product was also identified in non-FESLF–associated isolates from other parts of the world (Figure 8). This may indicate either that *ypmA* is not responsible alone for the scarlet-like symptoms, but it may be necessary in association with other genes, or that Russian and non-Russian isolates harbor different alleles with different activities. Most other Y. pseudotuberculosis IP31758 specific chromosomal genes were detected in several Y. pseudotuberculosis isolates of worldwide origins (Figure 8). Some of these genes have metabolic functions (periplasmic glucans biosynthesis gene *mdoG* or glycerol phosphate transporter *glpT*) and likely contribute to the overall bacterial fitness for the survival of Y. pseudotuberculosis in the environment. The small conjugative plasmid pYpsIP31758.2 was exclusively found in Y. pseudotuberculosis IP31758, which argues against a role of this plasmid during FESLF infection. Nevertheless, the encoded adhesive pilin structure may contribute to the Y. pseudotuberculosis IP31758 strain-specific FESLF symptoms, and its conjugal transfer apparatus may interact with the coharbored pYpsIP31758.1 plasmid and facilitate its transmission and spread. Interestingly, the only non-Russian strain that carries pYpsIP31758.1 also harbors ten of the 12 IP31758-specific genes. This strain was isolated from the biopsy of an otter in Sweden (Figure 8). It may thus be speculated that derivatives of FESLF-associated Y. pseudotuberculosis isolates are spreading among wildlife in this part of the globe, and that human cases of FESLF may appear in previously unscattered countries neighboring Russia.

### Genomic Plasticity and Pathogenicity in *Y. pseudotuberculosis*

The genome sequence comparison of two Y. pseudotuberculosis strains gives insights into the evolution of this important species and refines our understanding of genome reduction by lowering previous estimates of the number of genes lost in Y. pestis since emerging from *Y. pseudotuberculosis.* The genetic traits predicted to contribute to pathogenicity in Y. pseudotuberculosis IP31758, including two novel plasmids, comprise the majority of the strain-specific gene pool. We have presented evidence demonstrating that most of the unique genes in each sequenced Y. pseudotuberculosis strain were laterally acquired, and not lost in the other *Yersinia* as previously thought*.* By reducing the Y. pseudotuberculosis species-specific gene pool to 67, the number of putative genes lost in Y. pestis during the speciation process is also reduced (128 genes were found to be unique to Y. pseudotuberculosis IP32953 and Y. pestis CO92 [22]). Unlike the Y. pestis lineage that has undergone gene loss [22], our analysis indicates that lateral gene acquisition is the predominant driver in the evolution of Y. pseudotuberculosis species. In the case of Y. pseudotuberculosis IP31758, its unique gene pool was mainly acquired from Enterobacteriaceae and other soil-dwelling bacteria (Figure S2). The acquisition of a short DNA segment in a single event, such as observed for the inserted superantigenic toxin YPM or genes introduced by the novel plasmids pYpsIP31758.1 and pYpsIP31758.2, may be a major evolutionary step in the evolution of a species and sufficient to transform a pathogenic bacterial strain into a more severe variant, causing a drastically different disease, regardless of the preexisting chromosomal background heterogeneity. The Y. pseudotuberculosis IP31758 genome contains only 21 degenerate genes, which is far less than reported for the published Y. pestis genomes [23–26]. Driven by different environmental selective pressures, the two sequenced Y. pseudotuberculosis isolates appear to have undergone niche specific microevolution that led to two different strains with vastly different pathogenic potential and unique physiological capabilities.

## Materials and Methods

### Bacterial strains.

Y. pseudotuberculosis IP31758 (serotype O:1b) was isolated in 1966 from the stools of a patient presenting with scarlet-like fever in the Primorski region of the former Soviet Union and was sent in 1971 to the Institut Pasteur (Paris, France) by Dr. Timofeeva (Antiplague Institute, Irkoutsk, Russia). The strain sequenced and analyzed in this study was subcultured once from that original 1971 stock culture for the purpose of this study. A collection of 46 geographically and phenotypically diverse Y. pseudotuberculosis and Y. pestis strains was screened for the presence or absence of 18 loci specific to Y. pseudotuberculosis IP31758 (Figure 8).

### Genome sequencing and annotation.

Genomic DNA of Y. pseudotuberculosis IP31758 was subjected to random shotgun sequencing and closure strategies as previously described [103]. Random insert libraries of 3–5 kb and 10–12 kb were constructed, and 61,634 high-quality sequences of 837 nt average read length were obtained. A draft genome sequence was assembled using the Celera assembler [104]. An estimate of the copy number of each plasmid was obtained by dividing the coverage depth of the plasmid by the coverage depth of the chromosome. The chromosome and the two plasmids were manually annotated using the TIGR Manatee system (http://manatee.sourceforge.net).

### BLAST score ratio analysis.

For each of the predicted proteins of Y. pseudotuberculosis IP31758, a BLASTP raw score was obtained for the alignment against itself (REF_SCORE) and the most similar protein (QUE_SCORE) in each of the genomes of Y. pseudotuberculosis IP32953 and *Y. pestis* CO92. These scores were normalized by dividing the QUE_SCORE obtained for each query genome protein by the REF_SCORE. Proteins with a normalized ratio of <0.4 were considered to be nonhomologous. A normalized BLAST score ratio of 0.4 is generally similar to two proteins being 30% identical over their entire length [36].

### Screening analyses.

The primer pairs are listed as supporting information in Table S7. PCRs were performed with 1 U of *Taq* polymerase (Roche, http://www.roche.com) in the supplied buffer. PCR amplification reaction mixtures contained 10 μM of each primer and 1 mM dNTPs. The PCR program involved one step at 94 °C for 5 min, followed by 35 cycles of amplification of three steps: (1) 94 °C for 30 s, (2) 60 °C for 30 s, and (3) 72 °C for 7 min. PCR products were maintained at 72 °C for 7 min, separated by gel electrophoresis in 1% agarose gels, and stained with ethidium bromide.

### Genome visualization.

The chi squares and GC skews were computed according to Nelson et al. [103]. For the chromosomal chi square, a window size of 2 kb and a sliding window of 1 kb was used, while a window size of 1 kb and a sliding window of 0.2 kb were used for the two plasmids. GC skews were calculated using a window size of 1 kb for the chromosome and 0.2 kb for the two plasmids. The whole-genome alignment tool NUCmer [105] was used to calculate the overall gene identities to the respective Y. pseudotuberculosis and Y. pestis strains.

### Taxonomy BLAST.

Each of the 4,164 Y. pseudotuberculosis CDSs (not including the RNA genes) was blasted using BLASTP against the National Center for Biotechnology Information (NCBI) protein database (E-value > 10^−5^). The BLAST output was parsed using a custom Perl script that recorded the taxonomic affiliation of the BLAST best hit for each protein.

## Supporting Information

Figure S1 Circular Representation of the Y. pseudotuberculosis IP32953 Genome From outer to inner circle. Circle 1 and 2 predicted open reading frames encoded on the plus (circle 1) and minus strands (circle 2), colored according to the respective TIGR role IDs (http://cmr.tigr.org/tigr-scripts/CMR/RoleIds.cgi). (Circle 3) Noncoding RNAs. Brown, tRNA genes; black, ribosomal rRNAs. (Circle 4) GC skew. (Circle 5) Genomic islands. Violet, species-specific R/M system shared between Y. pseudotuberculosis strains IP31758 and IP32953. (Circle 6) IS elements, transposases, and phage integrases. (Circles 7–13) Comparative analysis of the genomic inventory. Intraspecies comparison to Y. pseudotuberculosis IP31758 (circle 7) and interspecies comparison to Y. pestis CO92 (circle 8), KIM (circle 9), 91001 (circle 10), Nepal516 (circle 11), Antiqua (circle 12), and Pestoides F (circle 13). (Circle 14) Chi square.
(8.6 MB AI).

Figure S2 Phylogenetic Analysis and Taxonomy BLAST of the Complete Genomic Inventory of Y. pseudotuberculosis IP31758 Each of the 4,164 Y. pseudotuberculosis CDSs except the noncoding RNAs were blasted using BLASTP against the NCBI protein database (E-value < 10^−5^).
(A) Taxonomic distribution of each protein. Almost 90% of the predicted Y. pseudotuberculosis proteins do have their closest homolog in the genus *Yersinia,* and only a minority of 3% in other microbes. For 9% of the genes, no database homologs were found.
(B) Gene acquisition via horizontal gene transfer. The majority of these genes group phylogenetically in the gamma-subdivision of proteobacteria, in particular into the family of Enterobacteriaceae.
(505 KB PPT)

Table S1 Comparative Analysis of the Restriction Profiles of pVM82 and pYpsIP31758.1 (35 KB XLS)

Table S2 Statistics of the Intra- and Interspecies BLAST Score Ratio Analyses (32 KB DOC)

Table S3 Species-Specific Genes in Y. pseudotuberculosis
(40 KB XLS)

Table S4 Strain-Specific Genes in the Y. pseudotuberculosis IP31758 Genome (68 KB XLS)

Table S5 Strain-Specific Genes in the Y. pseudotuberculosis IP32953 Genome (70 KB XLS)

Table S6 Genomic Regions Generating Genomic Plasticity in the Y. pseudotuberculosis IP31758 Genome (68 KB DOC)

Table S7 Primer Pairs (56 KB DOC)

### Accession Numbers

The sequences have been deposited in GenBank (http://www.ncbi.nlm.nih.gov/Genbank) under accession numbers CP000720 (chromosome), CP000719 (pYpsIP31758.1), and CP000718 (pYpsIP31758.2). The genome assembly has been deposited in the NCBI Assembly archive (http://www.ncbi.nlm.nih.gov) under Assembly ID (AI) 1935, and all sequencing traces are available from the NCBI trace archive under Contig ID numbers 280084, 280085, and 280086.

## Abbreviations

CDS: coding sequence
FESLF: Far East scarlet-like fever
HPI: high pathogenicity island
IS: insertion sequence
nt: nucleotide
PAI: pathogenicity-associated island
R/M: restriction/modification
YAPI: *Yersinia* adhesion pathogenicity island
YPM: Y. pseudotuberculosis–derived mitogen
