# Supplementary material for: The Complete Genome Sequence of Yersinia pseudotuberculosis IP31758, the Causative Agent of Far East Scarlet-Like Fever
Source: PLoS Genet. 2007 Aug 31;3(8):e142. doi: 10.1371/journal.pgen.0030142 (PMC1959361; doi:10.1371/journal.pgen.0030142)

Supplementary Figure 1

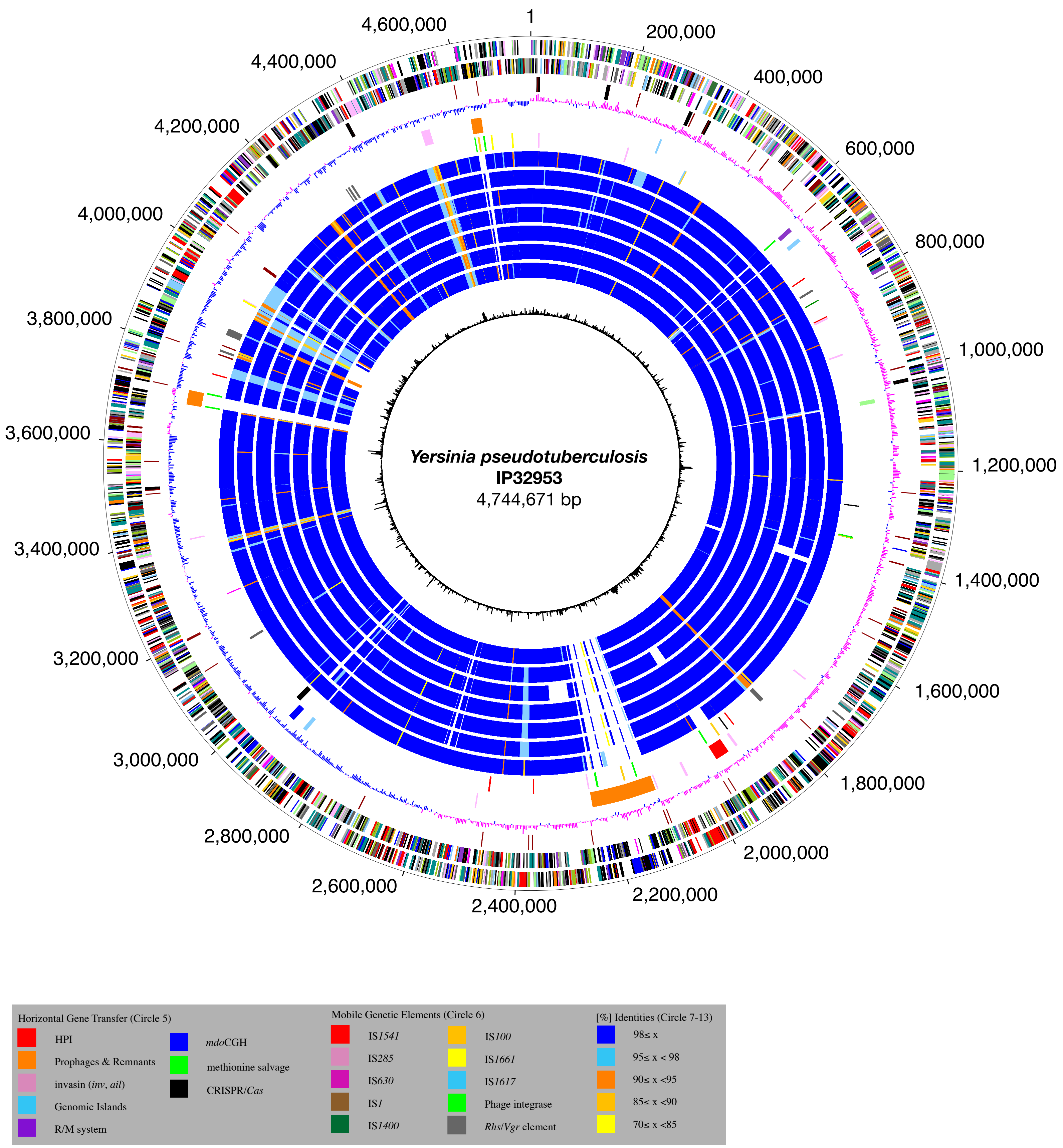

Supplement: Supplementary file 1 [file pgen.0030142.sg001.pdf]
