# Supplementary material for: The Complete Genome Sequence of Yersinia pseudotuberculosis IP31758, the Causative Agent of Far East Scarlet-Like Fever
Source: PLoS Genet. 2007 Aug 31;3(8):e142. doi: 10.1371/journal.pgen.0030142 (PMC1959361; doi:10.1371/journal.pgen.0030142)
Supplement: Supplementary file 2 [file pgen.0030142.st002.doc]

**Table S2.** **Statistics of the intra- and interspecies Blast Score Ratio analyses**

| Reference  Proteome | ***Y. pseudotuberculosis***  **IP31758** | | |
| --- | --- | --- | --- |
| Query  Proteomes | ***Y. pseudotuberculosis***  **IP32953** | ***Y. pestis***  **CO92** | ***Y. enterocolitica***  **8081** |
| Median | 0.9990 | 0.9968 | 0.8652 |
| Mean | 0.8979 | 0.8834 | 0.6531 |
| Standard Deviation | 0.2639 | 0.2659 | 0.3621 |
| Variance | 0.0696 | 0.0707 | 0.1311 |
