# Supplementary material for: The Complete Genome Sequence of Yersinia pseudotuberculosis IP31758, the Causative Agent of Far East Scarlet-Like Fever
Source: PLoS Genet. 2007 Aug 31;3(8):e142. doi: 10.1371/journal.pgen.0030142 (PMC1959361; doi:10.1371/journal.pgen.0030142)
Supplement: Supplementary file 3 [file pgen.0030142.st006.doc]

**Table S6. Genomic regions generating genomic plasticity in the *Y. pseudotuberculosis*** IP31758 genome

| **Designation** (Figure 1a) | **Genomic Region** | | **Coding Capacity** | **Putative DNA source, Taxonomic relation** |
| --- | --- | --- | --- | --- |
| **Phage remnant** | YpsIP31758_0044 | YpsIP31758_0054 | Enterobacterial phage integrase (YpsIP31758_0054), hypothetical proteins, conserved hypothetical proteins |  |
| **Genomic island** | YpsIP31758_0312 | YpsIP31758_0327 | Lipoprotein, hypothetical proteins | homology and synteny to *Enterobacteriaceae* PAIs |
| **Indel** | YpsIP31758_0669 | | Hypothetical protein | *Bacillus* species |
| **Imperfect duplication** | YpsIP31758_0726  YpsIP31758_0777  YpsIP31758_2511 | YpsIP31758_0733  YpsIP31758_0785  YpsIP31758_2503 | Rhs/Vgr1 element protein, OmpA, ATPase with chaperone activity, conserved hypothetical proteins, YpsIP31758_0726 to YpsIP31758_0733 is flanked by IS elements and downstream prophage elements | Genes are conserved in regards to synteny, although there are difference in coding frame and minor sequence differences (80-94% identity of individual genes at nucleotide level*)* |
| **Genomic island** | YpsIP31758_0743 | YpsIP31758_0777 | YAPI_IP31758_ phage integrase (YpsIP31758_0743, 100% nt identity to YpsIP31758_3686), phage and plasmid borne proteins (RepB), transcriptional regulators, hypothetical proteins | homology and synteny to *Enterobacteriaceae* PAIs |
| **Phage remnant (tRNA^Phe-1^)** | YpsIP31758_0806 | YpsIP31758_0809 | *Enterobacteriaceae* phage integrase (YpsIP31758_0809), conserved hypothetical proteins | *Enterobacteriaceae* |
| **Indel** | YpsIP31758_1282 | YpsIP31758_1285 | conserved hypothetical proteins. *Enterobacteriaceae* transposase **(**YpsIP31758_1282**)** |  |
| **Phage remnant (tRNA^Arg-2)^** | YpsIP31758_1383 | YpsIP31758_1386 | Hypothetical proteins |  |
| ***opg* locus** | YpsIP31758_1551 | YpsIP31758_1553 | Osmoregulated glucan biosynthesis (*mdo*CGH), *mdo*C degenerated, *Y. pseudotuberculosis* IP32953 (YPTB2493 to 2495) | Putative gene loss in *Y. pestis* lineage |
| **Indel** | YpsIP31758_1761 | | Hypothetical protein |  |
| ***ypmA* locus (*yrs)*** | YpsIP31758_1855 | YpsIP31758_1868 | Superantigenic YPMA toxin (YpsIP31758_1858) in instable chromosomal locus |  |
| **Prophage PhiYpsI (tRNA^Leu-2-Cys-1-Gly2^)** | YpsIP31758_2183 | YpsIP31758_2196 |  | Common phage genes of *Y.* *pseudotuberculosis* IP31758 and IP32953 (YpsIP31758_2186 to YpsIP31758_2196) |
| **Prophage PhiYpsII (tRNA^Leu-2-Cys-1-Gly2^)** | YpsIP31758_2197 | YpsIP31758_2250 | Enterobacterial phage structural and regulatory proteins, RelB/DinJ/StbE/Axe family addiction module antitoxins (YpsIP31758_2226, YpsIP31758_2227, YpsIP31758_2237, YpsIP31758_2238), hypothetical proteins | *Y. pseudotuberculosis* IP31758 strain-specific (YpsIP31758_2232 to YpsIP31758_2307) |
| **Indel** | YpsIP31758_2493 | YpsIP31758_2494 | Hypothetical proteins | *Y. pseudotuberculosis* IP31758 strain-specific |
| **Genomic island (tRNA^Thr-2-Ser-4^)** | YpsIP31758_2759 | YpsIP31758_2760 | TetR family transcriptional regulator, hypothetical protein | *Enterobacteriaceae* |
| **Prophage PhiYpsIII** | YpsIP31758_2930 | YpsIP31758_2946 | *Enterobacteriaceae phage* integrase (YpsIP31758_2946), structural phage proteins, hypothetical proteins | [*Burkholderia cenocepacia* phage BcepB1A](http://www.ncbi.nlm.nih.gov/Taxonomy/Browser/wwwtax.cgi?id=279530),  *Y. pseudotuberculosis* IP31758 strain-specific phage |
| ***mtn locus*** | YpsIP31758_3178 | YpsIP31758_3184 | Methionine Salvage Pathway (*mtn*KADCBE, methionine aminotransferase, amidohydrolase) | Putative gene loss in *Y. pestis* lineage |
| **R/M system, type I** | YpsIP31758_3536 | YpsIP31758_3538 | *hsd*MSH (methyltransferase, endonuclease, site-specific deoxyribonuclease) adjacent to phage integrase (YpsIP31756_3540) | Putative gene acquisition in *Y. pseudotuberculosis* IP31758 and IP32953 (YPTB0535-YPTB0537) |
| **YAPI_IP31758_ (tRNA^Phe-2^)** | YpsIP31758_3686 | YpsIP31758_3748 | pilin *pilWVUSRQPONML* cluster mediates pathogenicity | Enteropathogenic *Yersinia* genome pool, derivates in *Y. pseudotuberculosis* IP32777 and *Y. enterocolitica* 8081 |
| **Genomic Island** | YpsIP31758_4040 | YpsIP31758_4046 | Enterobacterial membrane protein, conserved hypothetical protein, hypothetical protein adjacent *Enterobacteriaceae* *IS*630 transposase (*E. coli*, *Shigella* species) (YpsIP31758_4040) | *Enterobacteriaceae* |
