# Supplementary material for: The Complete Genome Sequence of Yersinia pseudotuberculosis IP31758, the Causative Agent of Far East Scarlet-Like Fever
Source: PLoS Genet. 2007 Aug 31;3(8):e142. doi: 10.1371/journal.pgen.0030142 (PMC1959361; doi:10.1371/journal.pgen.0030142)
Supplement: Supplementary file 4 [file pgen.0030142.st007.doc]

Table S7. Primer pairs

| **ORF** | **Gene** | | **forward Primer (5’-3’)** | **reverse Primer (5’-3’)** |
| --- | --- | --- | --- | --- |
| YpsIP31758_0225 | *glpT* | | GATGTTCGTGCTGTTGTTCC | TAACCATAAAGCCACCGTCC |
| YpsIP31758_0759 | aec64 (predicted GTPase) | | AATGCGCGGAAAATAATGG | AGCGTTTGCATGTTTAGCG |
| YpsIP31758_0808 | *chp* (phage) | | TTTTCAAGGCCGCTTATCC | TTTTGCTGACTTAATCGTGGG |
| YpsIP31758_1552 | *mdoG* (glucan biosynthesis) | | TACTGGCGACATCAATCTGG | ATTGTCAGGAAAAGCAGCG |
| YpsIP31758_1858 | *ypmA* | | TGTTTTATCTGTTGGCTGCG | TTGCCGTAAGTTTGGAGAGC |
| YpsIP31758_2224 | *chp2224* (phage) | | TACAGAAAACTGGATCGCCC | TGGCGCAAAGGTAAAATCC |
| YpsIP31758_2931 | *gp* (*Burkholderia* BcepB1A) | | AACAAAAACGCCTGGTATGC | AACCCTTGGCTATCAACACG |
| YpsIP31758_3537 | *hsdS* | | AAACACCGAGCAAGAACTGG | ATTTGGTAACGGCTTTGGC |
| YpsIP31758_3686 | *api*95 (phage integrase) | | AACGCCGAGATTGAAAAGG | TGCCGTAAAAATGGTGCC |
| YpsIP31758_3734 | *pilN* | | ATGGTTTTCGTGCTCATCG | CATTCTCATTACCTGTTGGGC |
| YpsIP31758_3743 | *api*5 | | CTTGCAGGAAAACGGATACC | TTTGGAATGTTCTGCCACC |
| YpsIP31758_3748 | *api*1 | | ATCCTCCCATTTTCAACGG | TTCAGGCTCAGATTTTTCGG |
| YpsIP31758_A0038 | *inc*C | | ACGACGCCAATCAATAACG | CCTGGGTTTTCTTCTGTTCG |
| YpsIP31758_A0060 | *pilN* | | AAATGGAGTGGTGAAGTGCC | TTTCCTCTTTGCCTATTCGC |
| YpsIP31758_B0014 | *pilN* | AGTGGACAGAAAAACAGGGC | | TCAAACACACCTAACCAGCG |
| YpsIP31758_B0052/53 | *mucAB* (UV resistance) | TTACTTCTTGCGGGTATCGG | | TGTCGTTTATGGCAAAGGG |
| YpsIP31758_B0097 | *dotA* | GCAATGTTCTGATGTTTCGG | | ATACGCTCAAATCCTCCACG |
| YpsIP31758_B0121 | *dotO* | ATGATGGATGGTTCACTGGG | | ATTGATTGCTTGAGGTCTTGG |
